# Relationship Between Body Mass Index and the Incidence of Perineal Tears in Primigravid Women: A Retrospective Cross-Sectional Study at a Hospital in Mexico

**DOI:** 10.7759/cureus.104675

**Published:** 2026-03-04

**Authors:** Rashid Sadek Azar, Juan F Ramirez Roldan, Jose M Nuñez de la Vega

**Affiliations:** 1 Obstetrics and Gynecology, Hospital General de Especialidades, Campeche, MEX; 2 Epidemiology and Public Health, Hospital General de Especialidades, Campeche, MEX

**Keywords:** high body mass index, obstetric trauma, perineal tear, primigravid women, term vaginal delivery

## Abstract

Introduction: Perineal tears represent one of the main complications of vaginal delivery, with immediate and long-term consequences for maternal health. Although several factors have been associated with their occurrence, the role of maternal body mass index (BMI) remains controversial.

Objective: This study aims to evaluate whether there is a significant difference in the incidence of perineal tears among primigravid women with different BMI categories.

Methods: A retrospective cross-sectional study was conducted between March 2022 and February 2024 at the General Hospital of Specialties “Dr. Javier Buenfil Osorio” (Campeche, Mexico). A total of 180 (100%) primigravid women with term pregnancies were included and categorized according to BMI as normal weight, overweight, or obese. The incidence of perineal tears was compared among the groups.

Results: The overall prevalence of perineal tears was 56.7% (102 patients). Tear rates were similar across BMI categories: normal weight with 58.3% (35 patients), overweight with 51.7% (31 patients), and obese with 60.0% (36 patients). No statistically significant differences were found (p = 0.622). Additionally, no correlation was identified using Kendall’s Tau-b test (p = 0.854).

Conclusion: No significant association was observed between maternal BMI and the incidence of perineal tears in primigravid women. Other obstetric factors may play a more relevant role.

## Introduction

Perineal tears are injuries that occur in the perineum during vaginal delivery, either spontaneously or as a result of an episiotomy. These injuries may involve only the skin and perineal muscles or, in more severe cases, extend to the anal sphincter and rectal mucosa [1]. More than 85% of women who deliver vaginally experience some degree of perineal trauma, while third- and fourth-degree tears occur in approximately 0.6-11% of vaginal births [2-3]. Perineal tears have also been described as clinical indicators of levator ani muscle trauma, which can be identified by translabial ultrasound several months after delivery [4]. Severe perineal injuries are independently associated with levator ani avulsion and have been linked to an increased risk of long-term pelvic floor disorders [5].

Maternal body mass index (BMI) is known to influence pregnancy and delivery outcomes, including the risk of gestational diabetes, hypertensive disorders, cesarean delivery, and perineal trauma [6]. Women with elevated BMI are more likely to develop pregnancy-related complications that may increase the need for obstetric interventions, such as operative vaginal delivery or cesarean section [7]. However, the association between BMI and the risk of perineal tears remains unclear.

Several studies have suggested that obesity may reduce the likelihood of perineal trauma. Durnea et al. reported that women with obesity and severe obesity were less likely to experience mild perineal tears compared with women with normal BMI, although no significant association was observed with severe tears [8]. Similarly, Garretto et al. found a lower prevalence of third- and fourth-degree perineal tears among obese women, independent of parity, race, neonatal birth weight, or mode of delivery, raising the possibility of a protective effect of increased BMI [9].

In contrast, other authors have reported opposing findings. Frigerio et al. identified moderate to severe obesity as an independent risk factor for severe perineal tears, particularly when combined with instrumental delivery and increased fetal weight [10]. In a population-based study conducted in Bangladesh, Rahman et al. observed that overweight women had higher rates of pregnancy-related complications, including perineal tears, although their analysis was not limited specifically to perineal trauma [7].

Taken together, the available evidence underscores the complex and multifactorial nature of perineal injury. These findings suggest that maternal BMI alone is unlikely to be a reliable predictor of perineal tears and that its effect may depend on the presence of additional obstetric and intrapartum factors.

## Materials and methods

An observational, cross-sectional, retrospective study was conducted to compare the incidence of all perineal tears between women with a normal BMI (BMI <25 kg/m²) and those with a BMI of ≥25 kg/m². The study was carried out at the General Hospital of Specialties “Dr. Javier Buenfil Osorio” in Campeche, southeastern Mexico, between March 2022 and February 2024. Data were obtained from medical records.

Eligible participants were nulliparous women with singleton pregnancies, term gestation (≥37 weeks), maternal age between 18 and 34 years, cephalic presentation, and vaginal delivery. Exclusion criteria included multiple gestation, breech presentation, episiotomy, and known obstetric risk factors associated with an increased risk of obstetric anal sphincter injuries (OASIS) that could confound the analysis.

The independent variable was maternal BMI, calculated as weight in kilograms divided by height in meters squared (kg/m²). BMI was recorded during the first prenatal visit by either a nurse or an obstetrician. For the primary analysis, BMI was categorized according to the World Health Organization (WHO) criteria into three groups: <25 kg/m², ≥25 kg/m², and ≥30 kg/m², to facilitate clinical interpretation.

The dependent variable was the presence of perineal tears, defined as traumatic lacerations of the perineal tissues that occur during vaginal delivery as a result of stretching and mechanical stress on the vaginal outlet. These injuries were identified through immediate postpartum clinical examination of the vaginal and perineal region and documented in accordance with the International Classification of Diseases, 10th Revision (ICD-10) [11].

Extracted variables included maternal age, gestational age at delivery, weight, height, BMI, and the presence of perineal tears. Statistical analyses were performed using JASP® software version 0.18.3 (Released: 2024). Associations between variables were assessed using the chi-square test for independence and Kendall’s Tau-b coefficient for ordinal correlations. Statistical significance was defined as p < 0.05.

## Results

During the study period, 2,252 patients (100%) who underwent vaginal delivery were identified, of whom 1,311 patients (58.2%) met the inclusion criteria. These patients were further divided into three groups: normal-weight, overweight, and obesity groups. From each of these groups, 60 (33.3%) medical records were randomly selected for a total of 180 medical records.

Maternal age demonstrated a non-normal distribution (Kolmogorov-Smirnov test, p = 0.005), with a median of 21 years (range: 15-36 years). Gestational age at delivery also showed a non-normal distribution (p < 0.001), with a median of 39 weeks (range: 37-41.2 weeks). Overall, BMI had a median value of 27.0 kg/m² (range: 18.8-40.3 kg/m²). Median BMI values were 21 kg/m² in the normal-weight group (60 patients, 33.3%), 19.5 kg/m² in the overweight group (60 patients, 33.3%), and 22.5 kg/m² in the obesity group (60 patients, 33.3%) (Table 1).

**Table 1:** Distribution of maternal age, gestational age, and BMI BMI: body mass index; KS test: Kolmogorov-Smirnov test *The KS test was not performed since the overall sample did not achieve a normal distribution

	Age	Gestational age	BMI overall	Normal BMI	Overweight BMI	Obesity BMI
Minimum	15.0	37.0	18.8	15	15	15
25th percentile	18.0	38.0	23.2	19	18	19
Median	21.0	39.0	27.0	21	19.5	22.5
75th percentile	23.2	40.0	30.5	23	22	26
Maximum	36.0	41.2	40.3	28	34	36
KS test	0.1	0.1	0.1	*		
p-value	0.005	<0.001	0.04	*		

Among the 180 patients (100%) included in the analysis, perineal tears were identified in 102 patients (56.7%), while 78 patients (43.3%) did not present this complication (Table 2). When stratified by BMI category, perineal tears were observed in 35 patients (58.3%) in the normal-weight group, 31 patients (51.7%) in the overweight group, and 36 patients (60.0%) in the obesity group (Table 3).

**Table 2:** Perineal tears 0: no tear; 1: with tear

	Frequency	Percentage
0	78	43.3%
1	102	56.7%
Total	180	100%

**Table 3:** Perineal tears compared to weight O: observed; E: expected

Weight		Perineal tears				
		Yes		No		Total
Normal	O	35	58.3%	25	41.7%	60
	E	34	56.7%	26	43.3%	60
Overweight	O	31	51.7%	29	48.3%	60
	E	34	56.7%	26	43.3%	60
Obesity	O	36	60%	24	40%	60
	E	34	56.7%	26	43.3%	60
Total		102		78		180
Chi-square (df)		0.95 (2)				
p-value		0.62				

Inferential analysis using the chi-square test showed no statistically significant association between BMI category and the presence of perineal tears (χ² = 0.95, p = 0.62). This finding was further supported by Kendall’s Tau-b analysis (τ = 0.01, p = 0.85), indicating no correlation between maternal BMI and the incidence of perineal tears (Table 3).

## Discussion

From a clinical perspective, these findings emphasize the complexity of perineal trauma and the limitations of relying on a single explanatory factor. Maternal BMI, when assessed independently, does not appear to be a consistent predictor of perineal tears, a conclusion that aligns with much of the existing literature. Several authors have reported that intrapartum variables such as gestational age, length of labor, instrumental delivery, and fetal position are more closely associated with perineal injury than maternal body weight alone [12-13]. Along similar lines, Hu et al. highlighted the importance of tissue characteristics and obstetric management during the expulsive phase of labor, suggesting that these factors may have a greater influence on perineal outcomes than BMI itself [14].

At the same time, published data remain conflicting. Some studies have identified obesity as a contributing factor to perineal trauma, particularly in the presence of additional obstetric risk factors. Frigerio et al. reported an increased risk of severe perineal tears among women with moderate to severe obesity, especially when instrumental delivery was required [10]. In contrast, Yamasato et al. observed a lower prevalence of severe perineal tears in women with extreme obesity (BMI: ≥50 kg/m²), raising the possibility of a protective effect, although the biological mechanisms underlying this finding have yet to be clearly defined [15]. Taken together, these discrepancies suggest that BMI does not act in isolation and that its clinical relevance likely depends on the interaction with other maternal, fetal, and intrapartum variables.

Although the present study did not identify statistically significant differences between BMI categories, its clinical value lies in questioning the routine use of BMI as a standalone predictor of perineal trauma. Overreliance on maternal weight alone may oversimplify risk assessment and overlook other modifiable factors related to labor management and delivery technique.

The overall prevalence of perineal tears in this cohort was relatively high, affecting 102 patients (56.7%). This finding may reflect local population characteristics or institutional practices during childbirth and should be interpreted within that context.

Consistent with these observations, no significant association was found between maternal BMI and the incidence of vaginal perineal tears in this study. Tear rates were 58.3% (35 patients) in the normal-weight group, 51.7% (31 patients) in the overweight group, and 60.0% (36 patients) in the obesity group, with no statistically significant differences among groups (χ² = 0.95; p = 0.622). Kendall’s Tau-b analysis likewise showed no correlation between BMI category and perineal trauma (τ = 0.013; p = 0.854).

Overall, these findings support the null hypothesis that maternal body weight is not independently associated with the occurrence of vaginal perineal tears. This conclusion is consistent with the work of Blomberg, who reported that maternal obesity alone does not constitute a direct risk factor for perineal injury, although it may be associated with other obstetric complications [16]. The present study, therefore, adds to the growing body of evidence suggesting that perineal trauma should be approached from a multifactorial perspective rather than attributed to BMI alone.

Study limitations

This study has inherent limitations related to its retrospective design and reliance on medical record data. As the analysis was conducted at a single center, the findings may not be fully generalizable to other settings. In addition, the sample size and the absence of certain intrapartum variables may have limited the detection of subtle associations.

## Conclusions

No statistically significant differences or associations were identified among the variables analyzed, indicating that, within this clinical setting, none of them appears to act as an independent determining factor. These findings are in line with previous studies that have similarly failed to demonstrate a clear association, underscoring the multifactorial nature of the condition. Rather than providing definitive answers, the present results support the need to reconsider current assumptions and to explore alternative hypotheses in future research, including the evaluation of additional variables and the combined effect of multiple risk factors related to the pathology under study.
